# The Positive Loop at Work: A Longitudinal Long-Term Study of Transformational Leadership, Group Passion, and Employee Results

**DOI:** 10.3389/fpsyg.2021.726744

**Published:** 2021-10-28

**Authors:** Rosa Mindeguia, Aitor Aritzeta, Alaine Garmendia, Ainara Aranberri

**Affiliations:** ^1^Department of Basic Psychological Processes and Development, Faculty of Psychology, University of the Basque Country, San Sebastian, Spain; ^2^Department of Mechanical and Industrial Production, Mondragon Unibertsitatea, Mondragón, Spain

**Keywords:** leadership, positive emotions, affect, longitudinal study, proactive behavior

## Abstract

Positive psychology and positive organizational behavior studies recognize that leadership is extremely important for generating positive well-being. Despite the frequently reported significant positive correlations, the causal long-term relationship between leadership, positive high intense affect, and employee results remains unclear. The main objective of this study was to analyze the long-term (longitudinal) relation of transformational leadership and positive high-intensity emotions with employee group satisfaction, commitment, and proactive behavior. We built a longitudinal structural equation model to test a mediation model with two time points; 2,480 workers from 166 work units completed questionnaires at both time points. Our results reveal that positive high-intensity emotions mediate the relation between transformational leadership and proactive behavior of workers, the bidirectional relations between the variables were also analyzed. The present study is, to our knowledge, the first analyzing the long-term effect of TFL and collective high-intensity emotions on worker’s results longitudinally. Our findings reflect the great complexity of affect and affect-related results in organizations and highlight the need for more longitudinal research to clarify emotional processes at work.

## Introduction

Since the affective revolution in organizational research (Cameron et al., 2003), positive affect has attracted greater attention in organizational science with current researchers studying this state using new approaches and methods (Diener et al., 2020). Several conceptual and quantitative reviews have examined aspects of positive emotions and affect within organizational scholarship (e.g., Elfenbein, 2014; Ashkanasy and Dorris, 2017; García-Buades et al., 2020). Their findings highlight the importance of affectivity in organizational life, considering both antecedents and consequences of affect in the workplace at different levels of analysis.

In relation to this, Ashkanasy (2003) established a multilevel model of affect in organizations, in which leadership is posited as a social process that has a major effect on the moods and feelings of team members. Positive psychology and positive organizational behavior studies recognize the importance of leadership generating positive well-being (To et al., 2015). Given this, there have been calls for additional research on the relationship between positive leaders’ behavior, and positive employee outcomes, such as work engagement, satisfaction, and proactive behavior (Zhu et al., 2012).

Regarding the consequences of positive emotions at work, several different theories and frameworks have been used to understand how positive emotions produce positive outcomes in organizational contexts (Diener et al., 2020). In particular, positive affect has been related to outcomes such as creativity, individual and group satisfaction, commitment, and proactive work behavior (Ashkanasy and Dorris, 2017). In addition, research has suggest the importance of emotional competencies for wellbeing in organizations (Tesi, 2021). Nonetheless, most research on leadership and group emotions in organizations has relied on correlational and cross-sectional studies. Therefore, despite the frequently reported significant positive correlations, the causal relationship between leadership, positive affect, and employee behaviors and results remains unclear.

Furthermore, longitudinal studies of emotions and affect at work are based on short-term individual relationships such as day-to-day emotional changes in organizations (Diener et al., 2020). Such research has shown that work affective events (Weiss and Cropanzano, 1996), including leadership, are highly relevant for day-to-day affective reactions at work (Ohly and Schmitt, 2015). Nevertheless, less is known about longer-term relationship between work events and affect at work and it is important to investigate if the continuous exposure to positive and negative work events over time can also have an impact on employees’ affect (Casper et al., 2019).

Therefore, the main objective this study was to analyze the longitudinal relation of TFL and group positive high-intensity emotions with employees’ group satisfaction, commitment, and proactive behavior.

## Theoretical Background

### Transformational Leadership and Positive Emotions

TFL refers to a leadership style by which leaders motivate followers to identify with organizational goals and interests and to perform beyond expectations. According to Bass (1985), such leaders can inspire, motivate, and stimulate followers, communicating enthusiasm and vision, and exhibiting emotional competency (Bass, 1985). It is a multilevel construct that manifests functional characteristics at both individual and team levels (To et al., 2015). Research has confirmed that TFL has a significant impact on a different positive work outcomes at all the organizational levels (Wang et al., 2011).

Even of the literature has analyze different forms of positive leadership, as charismatic or servant leadership, in the present study we analyze the dimensions of TFL based on Rafferty and Griffin (2004) model. Transformational leadership is unique in terms of its strategic role toward organizational goals whereas for example servant leadership is focused on individual autonomy (Xie, 2020). In the present research we used TLF term in reference to employee’s perception of their leader’s behavior with four different dimensions: Positive leadership [referring to inspirational communication in the Rafferty and Griffin (2006) model], Vision, Supportive Leadership, and Goal emphasis dimension. Positive leadership dimension refers to the expression of positive and encouraging messages about the organization, and statements that build motivation and confidence (Rafferty and Griffin, 2004). Vision is defined as the expression of an idealized picture of the future based around organizational values. Supportive leadership refers to leader expressing concern for followers and taking account of their individual needs. And finally, we define the goal dimension as to have a clear vision of the future.

In the leadership literature, emotions are recognized as a crucial aspect of TFL (for a review, see Gooty et al., 2010). According to affective events theory (Weiss and Cropanzano, 1996), leaders generate affective events that influence teams positively or negatively, shaping the intensity and form of their emotional response, as reflected in their emotional or affective state.

Researchers in this field define affect as a general form of subjective feelings that incorporate longer-lasting but less intense moods, as well as more specific and intense emotions (To et al., 2015). As such, affect is usually described using two dimensions: valence and activation (Russell and Barrett, 1999). The present study focuses on positive affective states with high activation/intensity defined as passion and composed of emotions like enthusiasm, happiness, and pride (Lopez-Kidwell et al., 2018). TFL has been found to be more strongly associated with this emotional state than with others. Previous research showed that lower levels of TFL are related to an absence of a rewarding interaction rather than a presence of an aversive interaction, and hence, TFL is more strongly associated with positive affect than with negative affect (Tepper et al., 2018).

At the group level, a recent review reveals that leaders are a relevant source of positive affect, which disseminates among team members by an emotional contagion process (García-Buades et al., 2020). It has been recognized that leaders can arouse strong positive feelings in their followers (George, 2000; Dasborough and Ashkanasy, 2002), which, in turn, influence their work attitudes and behaviors (García-Buades et al., 2020). Moreover, a recent cross-sectional study showed that transformational behaviors of leaders influence positive high intensity and cohesion through team emotional intelligence of leaders (Mindeguia et al., 2021).

Transformational leaders attend to and support follower’s needs and help them to deal with stressors eliciting feelings of happiness and enthusiasm in their followers (Bono and Ilies, 2006) and in turn promoting positive affect. These leaders arouse enthusiasm and a passionate commitment to goals that followers may have previously perceived to be unimportant or impossible; in other words, TFL is an affective event that increases positive high-intensity emotions, which we define as passion (see Ilies et al., 2006).

*H1. TFL has a positive effect on passion in work units.*

### Positive Affect and Employee Results

The present study focuses on three specific group social resources that have been shown to be associated with group positive affect and performance (Diener et al., 2020). Satisfaction, commitment, and proactive behavior have been recognized as group resources that strengthen group performance (Peñalver et al., 2017).

It has been shown that TFL and positive affect enhance team performance (perceived and objective) through team goal commitment (i.e., motivated team members pursuing team goals), team satisfaction (i.e., team members being satisfied in terms of their team tasks and environments), and team helping behavior (i.e., team members exhibiting more helping behaviors) (Peñalver et al., 2017; García-Buades et al., 2020).

In their review on positive emotions at work, Diener et al. (2020) noted that affective states are already recognized as causal entities in workplace behavior. Nevertheless, they highlighted the need for more longitudinal studies in the field.

Cross-sectional research showed that positive group affective tone is likely to cause team members to focus on positive information about past experiences, resulting in a greater degree of certainty and confidence regarding the achievement of future team goals (Peñalver et al., 2017). Moreover, pleasant feelings lead members to consider pursuing team goals that are important and valuable, making them feel more committed to these goals (Seo et al., 2004).

Previous work based on affective events theory has demonstrated that emotions influence employees’ job satisfaction and Mostafa (2017) longitudinal study showed that positive affect had a positive relationship with job satisfaction. In the same vein, Wu and Wang (2015) found that TFL had a positive association with positive group affective tone, which in turn helps energize teams to be more proactive.

Taken together, these studies indicate that shared positive moods across work unit teams might influence the teams’ motivational (e.g., team goal commitment), attitudinal (e.g., team satisfaction), and behavioral (e.g., proactive behaviors) processes (García-Buades et al., 2020); and that positive high-intensity emotions could mediate the relationship between TFL and employee results (satisfaction, commitment, and proactive behavior).

*H2. Passion has a positive effect on proactivity in work units*.
*H3. Passion has a positive effect on satisfaction in work units.*
*H4. Passion has a positive effect on commitment in work units.*
*H5. TFL has an indirect effect on proactivity, satisfaction, and commitment mediated by passion.*

Nevertheless, taking into account the longitudinal design of our study, the possibility of a reverse association will also be analyzed. Though there is a paucity of data on reverse associations, it has been suggested that work events and affect are reciprocally related over time (Casper et al., 2019).

On the one hand, because positive and negative affect may influence recall and information processing (Bower, 1981), employees who experience positive affect at work might see work events as more positive and this may influence employees’ perception of work events that happen to them. It may be that follower affect influences the evaluation of the leader, and hence, work units with high levels of passion may also rate their leaders more highly (Dasborough and Ashkanasy, 2002; Barsade and Gibson, 2015).

On the other hand, research has proposed what are called positive feedback loops where positive emotions generate positive behaviors and outcomes that in turn feedback into positive emotions (Aknin et al., 2012). Therefore, we can suppose that outcomes such as proactive behavior, satisfaction, and commitment may also produce positive high-intensity affect.

## Materials and Methods

### Procedure

The current study was part of a larger research project on organizational management, which included 166 work units from 39 industrial organizations all of which are part of Mondragon Cooperative Corporation in the Basque Country (Northern Spain). In terms of size, 38.5% (*N* = 15) of the organizations can be considered small, 43.6% (*N* = 17) medium-sized, and 17.9% (*N* = 7) large. The 2,480 workers of the final sample were long term workers in different positions of the organizations.

Before data collection, we sought permission from the managers of all participating organizations. The participants respond the questionnaires in two ways, (randomly selected) via email or using the paper-and-pencil method (hard copy). The hard copy questionnaires were completed in large meeting rooms under the supervision of a human resources manager from the employees’ organization. All responses (both email and hard copy) were anonymous and data processing was performed in compliance with Spanish data protection law. The study was approved by the Research Ethics Committee of the Mondragon University.

We used a longitudinal design in which all variables were measured twice with 1 year lag between time 1 and time 2. The two data collection waves were between 1 and 3 years apart. At Time 1, 2,970 workers completed and returned the surveys; and of these respondents, 2,480 also completed and returned surveys at Time 2. In this sample (*n* = 2480), respondents had a mean age of 41 years and 65% were male. The data in both waves were aggregated to the group level with a final data set on 166 work units. No more descriptive information was presented due to the privacy agreement with the participating organizations.

The final model was constructed only at group level because of the agreement arrived with the participant organizations that established that individual data was excluded to publish.

### Measures

#### Transformational Leadership

We adapted Rafferty and Griffin (2006) scale for the Vision, Positive Leadership (inspirational communication in the original scale), and Supportive Leadership dimensions, adding the Organizational Culture Inventory (Cooke and Lafferty, 1983) for the Goal Emphasis dimension (“*My supervisor has a clear understanding of where we want our unit to be in 5 years*”). This scale was already used in previous studies showing a good validity (Mindeguia et al., 2021). Confirmatory factor analysis was then conducted to confirm the factor structure of the new scale. The model showed a good fit [χ^2^df = 227.48, *p* = 0.0001, confirmatory fit index (CFI) = 0.97, Tucker-Lewis index (TLI) = 0.96, root mean square error of approximation (RMSEA) = 0.06, 90%] with adequate factor loadings on four dimensions, replicating the structure of the original scale. The Cronbach’s alphas for the four dimensions (Vision, Positive Leadership, Supportive Leadership, and Goal Emphasis) were 0.85, 0.92, 0.93, and 0.86, respectively for Time 1 and 0.86, 0.90, 0.94, and 0.88 for Time 2.

#### Passion

The dimension considered for this construct is derived from Russell’s circumplex model of emotion classification (Russell, 1980). The “Passion” dimension (high intensity and pleasure) comprised four emotions (“In my work, I usually feel enthusiastic”). The Cronbach’s alpha obtained in the present study was 0.82 for times 1 and 2. The scale was already used in other studies (Mindeguia et al., 2021) showing the same structure at individual and group levels.

#### Job Satisfaction

Three items were used to assess this construct (Rafferty and Griffin, 2006). An example item is the following: “Overall, I am satisfied with my job.” This scale had an alpha of 0.89 for Time 1 and 0.88 for Time 2.

#### Proactive Behavior

The three items of the Individual task proactivity subdimension of the Positive Behavior scale were used in this study (Griffin et al., 2007). An example item is the following: *“Initiated better ways of doing your core tasks.”* This scale had an alpha of 0.93 for both Time 1 and 2.

#### Commitment

We used the Affective commitment to the organization factor of the Organizational Commitment Scale (Meyer et al., 1993). The Cronbach’s alpha was 0.80 for Time 1 and 0.82 for Time 2. An example item is the following: *“I really feel as if this organization’s problems are my own.”*

### Statistical Analysis

Data cleaning and descriptive data analyses were performed with IBM SPSS Statistics 22.0 (IBM Corp. Released, 2017). Relationships between the variables were analyzed with structural equation modeling in Mplus version 7.11 (Muthén and Muthén, 1998). Analyses were conducted in three steps. As a first step, the measurement models and the dimensionality of the latent variables at each time point were examined. As a second step, the measurement invariance over time was investigated for the latent variables. In the third step, structural models designed to explore the directional associations between the variables were specified and tested.

To determine if aggregating individual responses to team-level constructs is adequate, we followed the procedure described by Van Mierlo et al. (2009). That procedure includes the examination of rwg and ICC1 and 2. The rwg values are a measure of agreement within the group. ICC1 is the proportion of variance in ratings due to team membership, and ICC2 is the reliability of team mean differences (Klein et al., 2000). Bliese (2000) concluded that ICC1 values exceeding 0.05 are sufficient to warrant aggregation. LeBreton and Senter (2008) suggested cut-off values that range from 0.70 to 0.85 for ICC2. Also, they concluded that rwg values between 0.51 and 0.70 indicate moderate agreement; rwg values between 0.71 and 0.90 show strong agreement, and rwg values between 0.91 and 1.0 indicate strong agreement.

Due to the non-normality of the indicators observed, the robust maximum likelihood estimator was employed to determine model fit and magnitude of the relationships. To determine model fit, root mean square error of approximation (RMSEA), standardized root mean square error of approximation (SRMSEA), Tucker–Lewis Index (TLI), and comparative fit index (CFI) were estimated following Little (2013) recommendation. In this sense, RMSEA and SRMSEA values below 0.08 represent acceptable fit, CFI and TLI values between 0.90 and 0.95 represent reasonable model fit, and values above 0.95 represent excellent model fit (Brown, 2015).

Following Cole and Maxwell (2003) recommendations it is better to test the mediation model with two independent models instead to test the model with a unique structural equation. A unique model would not allow to establish causal relations in mediation models having only two time points (Little, 2013). Therefore, in testing our theoretical model, we used an autoregressive, cross-lagged design. This design has been recognized to be one of the strongest and least biased designs to assess mediation using two-time points (Maxwell and Cole, 2007).

To test the hypothesized model with two-time waves, the approach recommended by Cole and Maxwell (2003) was followed by testing two-wave mediation through two steps: first, testing the causal relationship between the predictor (TFL) at Time 1 and the mediator (Passion) at Time 2 controlling for the mediator at Time 1 (step 1); and second, testing the causal relationship between the mediator at Time 1 and the outcomes (proactive behavior, commitment, and satisfaction) at Time 2 controlling for the outcomes at Time 1 (step 2).

**FIGURE 1:**
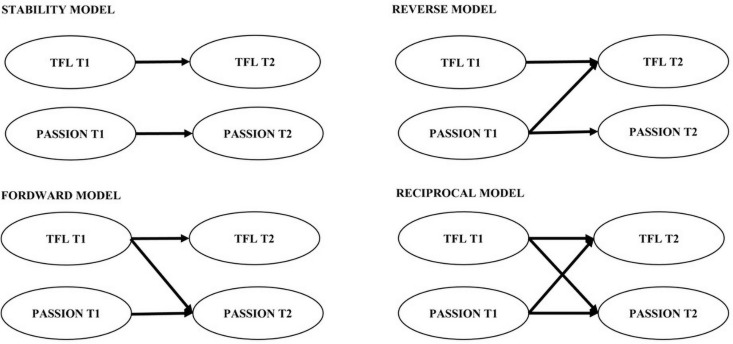
Simplified example of step 1 models. This is an example of the step 1 models, step 2 models follow the same structure. TFL, Transformational leadership.

For each step (1 and 2), four structural models were tested and compared: first, a stability model where every variable at Time 2 was predicted by the same variable at Time 1 (without cross-lagged associations); second, a forward model also including the hypothesized cross-lagged effects; third, a reversed model including cross-lagged effects that are opposite to the cross-lagged effects of the normal causation model; and finally, a reciprocal model combining the cross-lagged effects of the normal causation and reversed causation models. In all models, the error term of each Time 1 indicator was allowed to covary with the corresponding Time 2 indicator. For a better understanding, a representation of the four models for the step 1 is presented in Figure 1, the second step follow the same structure as step 1.

The magnitude of the mediation effect was estimated by multiplying the two cross-lagged paths (i.e., the path from TFL at Time 1 to passion at Time 2 and the path from passion at Time 1 to each groups’ outcomes at Time 2) and the significance of the mediation was assessed with Sobel test as recommended by Cole and Maxwell (2003) and Little (2013). Sobel test was used because there were two independent models (step 1 and step 2) and therefore bootstrapping could not be used (Preacher and Hayes, 2008).

## Results

### Measurement Model and Descriptive Analyses

For all the scales, ICC1 values were between 0.14 and 0.23, between 0.80 and 0.87 for ICC2, and between 0.69 and 0.70 for rwg therefore we concluded that the ICC1, ICC2, and rwg indices justified the aggregation of all variables in the study.

We ran confirmatory factor analyses to test metric invariance over time. We started with an unconstrained model (i.e., longitudinal configural invariance model) that included the factor models of all data collected in both data collection waves. The unconstrained model allowed for correlations between corresponding latent factors at Time 1 and Time 2 and between corresponding manifest variables (i.e., items) at both times. In the next step, we specified a constrained model (i.e., metric invariance model) in which we constrained the corresponding factor loadings to remain the same for each of our constructs over time. Finally, we specified a scalar invariance model by constraining the intercepts of items to be the same across groups. The results are shown in Table 1.

**TABLE 1:** Results of longitudinal invariance.

		Δ*CFI*	*CFI*	*TLI*	*RMSEA*	*SRMR*	Δχ*2(df)*
Configural invariance model	Passion	–	0.988	0.97	0.04	0.048	
	Transformational leadership	–	0.978	0.966	0.087	0.041	
	Satisfaction	–	0.998	0.993	0.030	0.024	
	Proactive behavior	–	1	1	0	0.022	
	Commitment	–	0.992	0.975	0.041	0.048	
Metric invariance model	Passion	0.003	0.985	0.98	0.060	0.070	1.79(3)
	Transformational leadership	0.003	0.975	0.957	0.088	0.090	18.93(2)
	Satisfaction	0.004	0.994	0.987	0.062	0.080	26.02(2)
	Proactive behavior	0.007	0.993	0.985	0.074	0.088	6.01(2)
	Commitment	0.008	0.984	0.966	0.082	0.086	14.05(2)
Scalar invariance model	Passion	0.004	0.981	0.970	0.066	0.062	1.71(3)
	Transformational leadership	0.013	0.962	0.936	0.087	0.089	15.77(2)
	Satisfaction	0.008	0.986	0.977	0.081	0.088	36.87(2)
	Proactive behavior	0.001	0.994	0.991	0.059	0.084	2.37(2)
	Commitment	0.002	0.986	0.977	0.067	0.081	1.37(2)

*The chi-square difference testing was corrected using Satorra-Bentler scaling correction (Muthén and Muthén, 2010).*

Given that RMSEA and SRMSEA are sensitive to model complexity (Van de Schoot et al., 2012), we considered that the fit indices were acceptable for all the variables. Moreover, in all models, at least two out of the three fit indices showed changes that were below the specified cutoff criteria. This suggests that the increasing equality constraints across the two samples specified in each of the subsequent models did not significantly worsen model fit, and hence, it can be concluded that the model showed acceptable invariance over time (Nelemans et al., 2019).

Descriptive statistics for all variables, including the means, standard deviations, and bivariate correlations between variables are shown in Table 2.

**TABLE 2:** Descriptive statistics and correlations between variables.

	**Mean (SD)**	**1**	**2**	**3**	**4**	**5**	**6**	**7**	**8**	**9**	**10**
1. TLF (t1)	4.09 (0.77)	–	0.61**	0.78**	0.70**	0.66**	0.51**	0.37**	0.36**	0.36**	0.39**
2. Passion (t1)	4.24 (0.61)		–	0.68**	0.49**	0.77**	0.22**	0.47**	0.20**	0.36**	0.32**
3. Satisfaction (t1)	4.50 (0.61)			–	0.58**	0.73**	0.32**	0.31**	0.30**	0.26**	0.26**
4. Proactive behavior (t1)	4.40 (0.60)				–	0.63**	0.41**	0.44**	0.32**	0.58**	0.40**
5. Commitment (t1)	4.19 (0.69)					–	0.23**	0.38**	0.23**	0.31**	0.40**
6. TLF (t2)	4.29 (0.77)						–	0.60**	0.79**	0.50**	0.72**
7. Passion (t2)	4.45 (0.51)							–	0.68**	0.52**	0.75**
8. Satisfaction (t2)	4.67 (0.49)								–	0.38**	0.78**
9. Proactive behavior (t2)	4.43 (0.62)									–	0.51**
10. Commitment (t2)	4.48 (0.62)										–

**p < 0.01. TLF, Transformational Leadership.

### Structural Equations

The fit indices of the models indicated that the reciprocal model provided a better fit to the data than the stability, forward, and reverse models in both steps.

**FIGURE 2:**
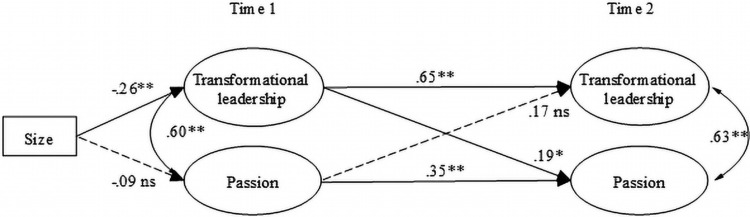
First part of the mediation model. ^∗∗^*p* < 0.01; ^∗^*p* < 0.05; ns, non-significant.

**FIGURE 3:**
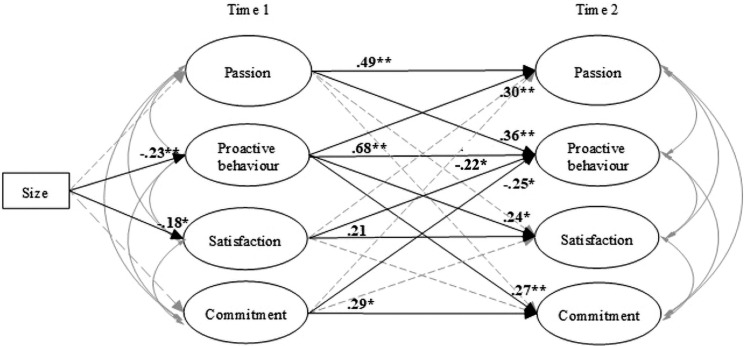
Second part of the mediation model. The paths in grey are non significants paths and correlations. **p < 0.01; *p < 0.05; ns, non-significant.

Generally, the results of the models indicate a reasonable fit: The reciprocal model of the step 1 (CFI = 0.94; SRMSEA = 0.06) showed an acceptable fit, and the reciprocal model of the step 2 (CFI = 0.98; SRMSEA = 0.05) a good fit. The path diagrams for both models are presented in Figures 2, 3. For clarity, the non-significant paths and the correlations between variables at the same time point were omitted from the figure. The non-significant regression coefficients are, however, reported in Table 3.

**TABLE 3:** Fit indices for proposed models.

*Model*	χ*2*	*df*	*RMSEA*	*TLI*	*CFI*	*SRMSEA*
*Cross-lagged relationships between transformational leadership and passion*						
Stability	19.70	4	0.15	0.82	0.93	0.10
Forward	16.953	3	0.17	0.78	0.94	0.10
Reverse	19.379	3	0.18	0.75	0.93	0.09
Reciprocal	15.70	2	0.20	0.70	0.94	0.06
*Cross-lagged relationships between passion and results*						
Stability	32.826	16	0.08	0.92	0.92	0.08
Forward	29.735	11	0.10	0.88	0.96	0.08
Reverse	32.477	8	0.14	0.78	0.95	0.08
Reciprocal	14.012	4	0.12	0.82	0.98	0.05

As can be seen in Figure 1, all the variables in the step 1 model showed high stability (β = 0.65 *p* < 0.01; β = 0.35 *p* < 0.01). The results indicate that TFL at Time 1 had a positive effect on Time 2 passion (β = 0.19 *p* < 0.05), and passion at Time 1 had a non-significant effect on Time 2 TFL. TFL and passion were significantly correlated at both times. Note that all the regression coefficients in the study are standardized.

Figure 2 shows the path model of the relations between passion, proactive behavior, satisfaction, and commitment (step 2). All variables except satisfaction (β = 0.21 ns) showed good stability. Passion at Time 1 showed a positive significant effect on Time 2 Proactive behavior (β = 0.36 *p* < 0.01) but not on other variables at Time 2. Proactive behavior at Time 1 showed a significant effect on Time 2 passion (β = 0.30 *p* < 0.01), satisfaction (β = 0.24 *p* < 0.05), and commitment (β = 0.27 *p* < 0.01). Further, satisfaction and commitment at Time 1 showed a negative significative effect on proactive behavior at Time 2 (β = −0.22 *p* < 0.05; β = −0.25 *p* < 0.05; respectively).

The mediation effect was estimated by multiplying the coefficient of the path from TFL to passion (β = 0.19) by that of the path from passion to proactive behavior (β = 0.36) (Cole and Maxwell, 2003; MacKinnon and Luecken, 2008), this yielding an effect of 0.25. A one-tailed Sobel test indicated that this mediation effect was significant (*z* = *1.92*, *p* < *0.05*). In order to confirm that this was a mediation effect, a reciprocal model was estimated, the relationship between TLF and proactive behavior confirmed that there was not a long-term direct relationship between the two variables. Therefore, H5 was partially supported as the relationship of TLF and proactive behavior was only by passion emotional state. The results are reported in Table 4.

**TABLE 4:** Direct effects and eliminated paths.

***Relationship***	***b***	***p value***
*Direct effect model TL and proactive behavior*		
TL_T1 to proactive behavior T2	–0.08	0.51
Proactive behavior T1 to TL T2	0.07	0.36
*Non-significant effects in proposed models (paths in gray)*		
*Control variables*		
Size to passion T1	–0.08	0.21
Size to commitment T1	–0.05	0.35
*Relationship between variables*		
Passion T1 to TL T2	–0.22	0.10
Passion T1 to satisfaction T2	0.01	0.97
Passion T1 to commitment in T2	0.07	0.57
Satisfaction T1 to passion T2	–0.14	0.20
Satisfaction T1 to commitment T2	–0.15	0.15
Commitment T1 to passion T2	–0.09	0.48
Commitment T1 to satisfaction T2	–0.08	0.67

*TL, Transformational leadership; T1, Time 1; T2, Time 2.*

## Discussion

This study sought to explore the causal relation between TFL, positive high-intensity group emotions (the emotional state passion), and workers’ results and wellbeing (satisfaction, commitment, and proactive behavior). Although a large number of studies have demonstrated effects of TFL on various organizational variables (Miao et al., 2016), only a few have analyzed effects of TFL and collective emotions through a long-term longitudinal study.

As we noted earlier, the long-term two-way relationships between leadership, emotions, and results of workers remain unclear. Our analysis supports the idea that, first, TFL has a positive effect on positive high-intensity emotions of work units, and second, positive high-intensity affect has a positive effect on proactivity in work units.

Analyzing the results separately, the first hypothesis stated that TFL has a positive impact on positive affect in work units. In relation to this, we can conclude that perceived transformational behaviors do enhance group positive affect. This result is in line with affective events theory, in the sense that TFL constitutes a positive affective event enhancing positive affect on workers (To et al., 2015). Regarding the reverse relationship, our results did not show a significant effect of positive emotions on TFL, in other words, they do not support the view that positive high-intensity emotions make workers give a better evaluation of TFL, as proposed by some authors (see, for example, Dasborough and Ashkanasy, 2002). Nevertheless, the time lag in our study could have been too long to detect such an effect, and hence, we cannot conclude there is no reciprocal relationship on shorter timescales.

Hypothesis two stated that passion would have a positive effect on proactive behavior in work units. Our model showed that a positive high-intensity affective state at the unit level produces more proactive behaviors in work units. This result is in line with those obtained in cross-sectional studies, such as that of Wu and Wang (2015) who found that leaders were able to promote team proactivity by cultivating a positive affective tone within teams, reflecting an energizing process in motivating proactivity. Moreover, our findings confirm that passion mediates the effect of TFL on proactive behavior.

Further, we observed a bidirectional association between positive high-intensity emotions and passion. This result is in line with the concept of positive feedback loops, in which positive emotions generate positive behaviors and outcomes that in turn feedback into positive emotions (Aknin et al., 2012).

Hypotheses three and four stated that passion has a positive effect on satisfaction and commitment in work units. Surprisingly, we did not find a significant relationship between passion and worker satisfaction and commitment. Overall, not much is known about the ideal time lags in occupational health research (Taris and Kompier, 2003). Nevertheless, previous longitudinal research, such as that of Mostafa (2017), has found evidence of a causal effect of positive emotions on satisfaction with a 6-month time-lag. Therefore, our findings could be explained by the time lag in the study, which may be too long to analyze this relationship.

The results of the present study deviated markedly from what we hypothesized at first and reflect the complexity of organizational life. In particular, the structural longitudinal models showed that the relation of emotions and proactive behaviors of work units is bidirectional. Moreover, we observe a bidirectional relationship between proactive behavior, satisfaction, and commitment in work units.

The results showed that proactive behavior has a positive effect on passion, satisfaction, and commitment in work units. This helps to confirm the positive loop thesis (Aknin et al., 2012) mentioned before, in which proactive behavior of work units positively influences high-intensity positive emotions, satisfaction and commitment among workers. At the same time, satisfaction and commitment have a negative effect on proactive behavior in work units.

Bakker and Oerlemans (2010) of subjective well-being in organizations suggested that proactive behaviors are more likely when there is a combination of high activation and high pleasure. Job satisfaction and commitment reflect only low-to-moderate levels of activation (and high pleasure) and imply a cognitive evaluation of one’s job, which taken together may not be enough to enhance performance. Employees who are satisfied with their jobs experience high pleasure but may have limited energy or aspirations (Grebner et al., 2005). In this sense, the positive response to items such as “Overall, I am satisfied with my job” (example from the satisfaction scale) does not indicate high activation. Low-intensity related results may be connected to low-intensity positive affect and lead to positive results and wellbeing in workers; nevertheless, such a low intensity does not produce activation (Bakker and Oerlemans, 2010). Based on that, the activation of worker’s well-being factors (as proactive behavior, commitment and satisfaction) could explain not only the intensity of affect but also the negative effect of satisfaction and commitment on proactive behavior and the lack of effect of passion on these factors. Nevertheless, more reasons could explain the results as the time lag of the study and future studies should analyze them carefully. For the inverse relationship (from proactive behavior to satisfaction) our results follow the already established theories that showed that forms of proactive behaviors as job crafting are positively related to satisfaction and commitment (Demerouti, 2014).

The present study is, to our knowledge, the first analyzing the long-term effect of TFL and collective high-intensity emotions on worker’s results longitudinally. Our findings reflect the great complexity of affect and affect-related results in organizations and highlight the need for more longitudinal research to clarify emotional processes at work.

This study contributes to this field in that it helps to clarify the reciprocal relations between the variables analyzed and the importance of the intensity, rather than quality, of emotions and affect-related characteristics in organizations. As posited by To et al. (2015), while affective valence has traditionally been regarded as the most influential dimension of job-related affect, affective activation also plays an important role in motivating job behaviors.

### Practical Implications

Our findings have several implications. First, they highlight the importance of emotions and affectivity at the group level. Although the idea that positive affect has a relevant role in organizations is not new (Barsade and Gibson, 2015), our data underline that organizations should care about and focus on employees’ emotions, as well as group emotions. Leaders need to effectively manage the cognitive characteristics of team members, but also their emotional responses, as these positively influence organizational outcomes (Ashkanasy et al., 2002). Further, we suggest that organizations should try to foster positive high-intensity affect in their employees, for instance, by promoting TFL behavior in managers (Breevaart et al., 2014).

Nevertheless the problem of actual TFL training program is that interventions are usually directed to a single source (employees or leaders). This interventions present some problems: The strategy that focuses only on the self-perception of leaders, allows acquiring knowledge for the training of these leaders since we work with them from their point of view. However, leaders are not always perceived as they think they are, and their positive or negative effect depends largely on how workers perceive them (REF). Contrary to that, if the research and intervention are based solely on the perception of the subordinates, the implementation of the acquired knowledge may not be effective (since it does not have the point of view of the leaders).

Therefore, we propose that interventions should emphasize the variables that make the link between the perception and intention of the directors during the trainings as emotional competencies. Training emotional competencies, both individually and as a group, could be a key element for creating positive loops in the company and therefore a healthy organization (Ashkanasy and Dorris, 2017). Through it, both, managers, and workers, learn to manage the group’s emotions as well as to better relate to their environment.

### Limitations and Future Directions

Our study has certain limitations that need to be considered. First, the results are based on self-report data and they may be affected by social desirability bias. It would also be useful to examine emotions and their relationship to performance in different cultural contexts and different kinds of projects. In this respect, the fact that we examined the hypothesized relationships within a single organizational context limits the generalizability of the findings. A related issue to consider here is that all the organizations included in this study were cooperatives, whose characteristics and functioning differ considerably from those of other types of companies. Future studies should therefore explore the relationships observed in different organizational contexts.

Further, even though this study had a longitudinal design, we only used two time waves to analyze a mediation effect. As we mentioned earlier, half-longitudinal designs are better for studying mediation effects than pure cross-sectional designs (Maxwell et al., 2011), but they are still vulnerable to bias. Future research should analyze the mediation effect using a longitudinal design with at least three time waves.

Another limitation to note is that we only considered emotions classified as high-intensity positive emotions, those that could be expected to have the strongest effect, based on the literature. A task for future research would therefore be to investigate the impact of other types of emotion on the process of leadership.

Finally, it is worth mentioning that we did not examine gender differences in TFL, and this may be relevant since the leadership teams in our sample were not homogeneous in this respect. About 30% of teams were comprised solely of men, while the others had one or more female members; there were no women-only leadership teams. In light of recent findings in this context (Hackett et al., 2018), future studies should examine whether the gender composition of teams influences the mediation effect observed here.

Another limitation with the TFL concept is that we do not analyze the different dimension of TFL separately. In this sense, Van Knippenberg and Sitkin (2013) recommend the study of specific dimensions of leadership, nevertheless, we could not analyze the separate dimension due to the sample size. Therefore, we recommend for future lines to analyze the dimensions separately to analyze better the effect of leaders behavior.

Despite these limitations, our study provides interesting empirical and longitudinal results and adds to knowledge about the influence of emotions on organizations and effective leadership. More specifically, it highlights the need for organizations to focus not only on promoting TFL styles within their management teams but also on eliciting high-intensity positive emotions in their followers. In other words, they achieve effective leadership which enables them to become healthy as well as productive organizations.

## Data Availability Statement

The raw data supporting the conclusions of this article will be made available by the authors, without undue reservation.

## Ethics Statement

The studies involving human participants were reviewed and approved by the Mondragon University Ethics Committee. The patients/participants provided their written informed consent to participate in this study.

## Author Contributions

AtA and RM were responsible of developing the theoretical foundations of the manuscript (Introduction, Discussion, and Conclusion). RM, AG, and AnA were responsible of the methodological part of the manuscript and especially of the statistical analysis. AG was responsible of the process for gathering data and reviewing the manuscript. All authors contributed to the article and approved the submitted version.

## Conflict of Interest

The authors declare that the research was conducted in the absence of any commercial or financial relationships that could be construed as a potential conflict of interest.

## Publisher’s Note

All claims expressed in this article are solely those of the authors and do not necessarily represent those of their affiliated organizations, or those of the publisher, the editors and the reviewers. Any product that may be evaluated in this article, or claim that may be made by its manufacturer, is not guaranteed or endorsed by the publisher.
